# Machine Learning-Driven QSAR Modeling of FXIa Inhibitors for Virtual Screening and Rational Drug Design

**DOI:** 10.3390/ph19060912

**Published:** 2026-06-10

**Authors:** Ali Onur Kaya, Mert Can Emre, Nesrin Emre

**Affiliations:** 1 Radiotherapy Department, Health Services Vocational School, Akdeniz University, 07070 Antalya, Türkiye; 2 Motor Vehicles Department, Sorgun Vocational School, Yozgat Bozok University, 66000 Yozgat, Türkiye; m.can.emre@yobu.edu.tr; 3 Department of Mathematics and Science Education, Faculty of Education, Akdeniz University, 07070 Antalya, Türkiye; nemre@akdeniz.edu.tr

**Keywords:** quantitative structure–activity relationship (QSAR), machine learning (ML), FXIa inhibitors, SHAP analysis, applicability domain, drug design

## Abstract

**Background/Objectives**: Coagulation factor XIa (FXIa) has emerged as a promising therapeutic target for the development of safer anticoagulant therapies with reduced bleeding risk. This study aimed to develop an interpretable machine learning-driven quantitative structure–activity relationship (QSAR) framework for predicting the inhibitory activity of FXIa inhibitors and supporting virtual screening applications. **Methods**: A total of 3026 curated compounds retrieved from the ChEMBL database were used for regression modeling, whereas 2119 compounds were retained for classification modeling after excluding intermediate-activity molecules. Molecular descriptors were generated using RDKit, Mordred, and Morgan fingerprint representations. Following preprocessing and feature selection, multiple machine learning algorithms were systematically benchmarked. Model robustness and reliability were further evaluated using 5-fold cross-validation, scaffold-aware validation, applicability domain analysis, and Y-randomization testing. **Results**: Nonlinear ensemble learning approaches consistently outperformed conventional linear algorithms. The optimized HistGradientBoostingRegressor achieved the best regression performance, with an independent test-set R^2^ value of 0.711 and an RMSE value of 0.759, whereas the optimized classification model achieved accuracies approaching 95%. SHAP analysis identified lipophilicity-related descriptors, aromatic scaffold organization, electrostatic surface properties, and molecular topology as major contributors to FXIa inhibitory activity prediction. In addition, a proof-of-concept virtual screening workflow successfully identified several candidate compounds exhibiting high predicted pKi values and elevated active-class probabilities. **Conclusions**: The proposed framework provides a robust, interpretable, and reproducible machine learning-driven QSAR strategy for FXIa inhibitor discovery and may facilitate future virtual screening campaigns and medicinal chemistry optimization studies targeting FXIa-associated anticoagulant drug discovery.

## 1. Introduction

Cardiovascular diseases remain the leading cause of mortality worldwide, and thrombosis plays a central role in the development of life-threatening conditions, such as myocardial infarction and ischemic stroke. Consequently, anticoagulant therapy is an essential component of modern cardiovascular treatment strategies. However, the currently available anticoagulants targeting thrombin and factor Xa are frequently associated with an increased risk of bleeding complications, which substantially limits their long-term safety and clinical applicability [1,2].

In this context, coagulation factor XIa (FXIa) has emerged as a promising therapeutic target because FXI contributes significantly to thrombus formation while playing a comparatively limited role in physiological hemostasis [1,2,3,4]. These unique biological characteristics have generated increasing interest in FXIa inhibition as a strategy for reducing thrombotic risk without markedly increasing bleeding tendency. Clinical and preclinical studies further support this concept, suggesting that FXIa inhibitors may represent a new generation of safer anticoagulant therapies [5,6]. Recent clinical candidates, such as milvexian and asundexian, highlight the growing therapeutic importance of FXIa-targeted anticoagulant drug discovery.

The FXIa active site contains multiple binding subsites that collectively create a structurally complex interaction environment governed by steric, hydrophobic, aromatic, and electrostatic interactions. These physicochemical characteristics strongly influence ligand recognition, selectivity, binding orientation and inhibitory potency. Structural and crystallographic studies have demonstrated that even subtle variations in ligand orientation or pocket occupancy may substantially alter FXIa binding affinity, emphasizing the importance of understanding detailed structure–activity relationships (SARs) for the rational design of potent FXIa inhibitors [7,8].

Quantitative structure–activity relationship (QSAR) modeling has long served as an important computational strategy for correlating molecular structure with biological activity and for predicting compound potency prior to experimental evaluation [9]. Traditional QSAR approaches are generally based on linear statistical methods and have provided valuable insights into the molecular determinants governing biological activity. Nevertheless, linear QSAR models often fail to adequately describe the highly complex, nonlinear, and multidimensional relationships associated with biochemical recognition processes and medicinal chemistry datasets [9,10].

Recent advances in machine learning (ML) and the rapid expansion of large-scale bioactivity databases, such as ChEMBL, have substantially enhanced the predictive potential of QSAR modeling [10,11]. In particular, ensemble-based machine learning architectures, including Random Forest and gradient-boosting approaches, have demonstrated strong predictive capabilities across diverse drug discovery applications involving chemically heterogeneous datasets [11,12,13]. Despite these advances, one of the major limitations of many ML-based QSAR frameworks is their limited interpretability, as numerous predictive systems still operate as “black-box” models. This limitation restricts the ability of medicinal chemists to rationally interpret the molecular determinants governing biological activity and consequently hinders mechanism-guided drug optimization. To address this issue, explainable artificial intelligence (XAI) approaches, such as SHapley Additive exPlanations (SHAP), have increasingly been applied to quantify descriptor contributions and improve the mechanistic interpretability of machine learning predictions [13,14].

To date, most computational studies on FXIa inhibition have primarily relied on structure-based approaches, including crystallographic analysis, molecular docking, virtual screening, and molecular dynamics simulations, often using relatively small datasets. In contrast, relatively few studies have developed large-scale, interpretable, and rigorously validated QSAR frameworks specifically designed for FXIa-inhibitor prediction. Furthermore, rigorous validation procedures, including applicability domain (AD) analysis, Y-randomization testing, and scaffold-aware validation, have not been consistently incorporated, despite their critical importance for evaluating model reliability and predictive generalizability toward chemically distinct compounds [14,15,16,17,18].

In this study, we developed a comprehensive and interpretable machine learning-driven QSAR framework for predicting FXIa inhibitory activity using a large curated ChEMBL-derived dataset containing structurally diverse inhibitor chemotypes. The proposed workflow integrates large-scale algorithm benchmarking, combined regression and classification modeling, scaffold-aware validation, applicability domain analysis, Y-randomization testing, hyperparameter optimization, and SHAP-based explainable artificial intelligence within a unified predictive framework. Furthermore, a proof-of-concept virtual screening workflow was incorporated to demonstrate the practical applicability of the developed models for hit prioritization in drug discovery. Collectively, the integration of predictive performance, mechanistic interpretability, and rigorous validation procedures enabled the development of a chemically meaningful and generalizable framework suitable for virtual screening, medicinal chemistry optimization, and rational design of structurally diverse FXIa inhibitors.

## 2. Results and Discussion

### 2.1. Dataset Curation and Descriptor Filtering

The initial FXIa bioactivity dataset retrieved from the ChEMBL database contained 5368 raw activity records, including experimentally reported Ki and IC_50_ measurements. Following canonical SMILES standardization and duplicate removal, the dataset was reduced to 3637 unique molecular structures. Additional molecular curation procedures, including the removal of invalid SMILES, elimination of incomplete structures, counterion filtering, and structural consistency control, further reduced the dataset to 3523 chemically valid compounds.

Subsequently, continuous activity-based filtering was applied to retain compounds with valid pKi values suitable for machine learning analysis. After activity standardization and regression-oriented filtering procedures, the final curated regression dataset consisted of 3026 FXIa inhibitors. This multistep curation workflow substantially improved the structural consistency and reliability of the dataset prior to descriptor generation and predictive modeling [19]. Molecular descriptor generation initially produced approximately 1826 Mordred descriptors representing the constitutional, topological, geometrical, electronic, and physicochemical properties of the molecules. Because large descriptor matrices may introduce redundancy, multicollinearity, and model instability, a structured descriptor-filtering workflow was applied, wherein descriptors with missing values, undefined outputs, or constant values were removed during the first filtering stage [20]. Correlation-based redundancy reduction was then performed to eliminate highly correlated descriptors and reduce multicollinearity within the descriptor space. Following descriptor pre-processing and redundancy reduction, the final machine-learning descriptor matrix consisted of approximately 309 descriptors. The large reduction from the initial descriptor space to the final filtered descriptor matrix indicates substantial redundancy within the raw Mordred descriptor pool. The final descriptor set provided a more compact, interpretable, and computationally efficient representation of the curated FXIa chemical space while preserving the structural and physicochemical diversity required for robust QSAR modeling [19,20].

### 2.2. Benchmarking and Regression Model Performance

To identify the most suitable machine learning algorithm for FXIa activity prediction, a comprehensive benchmarking analysis was conducted using multiple regression models under identical pre-processing conditions. The evaluated algorithms included both conventional linear approaches and advanced, nonlinear ensemble-based methods [21]. A comparative summary of the predictive performances of the evaluated regression algorithms is presented in Table 1.

The benchmarking results demonstrated substantial performance differences between the linear and nonlinear machine learning architectures. Conventional linear regression models, including LinearRegression, Ridge, BayesianRidge, and Lasso, exhibited relatively limited predictive capability, indicating that the relationship between molecular descriptors and FXIa inhibitory activity could not be adequately represented using simple linear relationships. In contrast, ensemble-based machine learning algorithms consistently demonstrated superior predictive performance. Among the evaluated models, the HistGradientBoostingRegressor achieved the highest predictive accuracy, with an independent test-set R^2^ value of approximately 0.71 and an RMSE of approximately 0.76. Additional high-performing models included ExtraTreesRegressor, LGBMRegressor, RandomForestRegressor, and Support Vector Regression (SVR), which also demonstrated strong predictive capabilities across the curated FXIa dataset. The superior performance of nonlinear ensemble-based approaches suggests that FXIa inhibitory activity depends on complex and nonlinear relationships among the physicochemical, topological, and electronic molecular features. Tree-based boosting and ensemble learning architectures are particularly effective in capturing multidimensional structure–activity relationships within curated descriptor spaces. The observed superiority of ensemble-based models over conventional linear approaches additionally supports the use of advanced machine learning methodologies for modeling heterogeneous medicinal chemistry datasets containing structurally diverse FXIa-inhibitor scaffolds [21,22,23]. These findings are consistent with previous reports demonstrating the effectiveness of ensemble learning strategies in ligand-based QSAR modeling and bioactivity prediction tasks.

### 2.3. Cross-Validation and Hyperparameter Optimization

**Figure 1:**
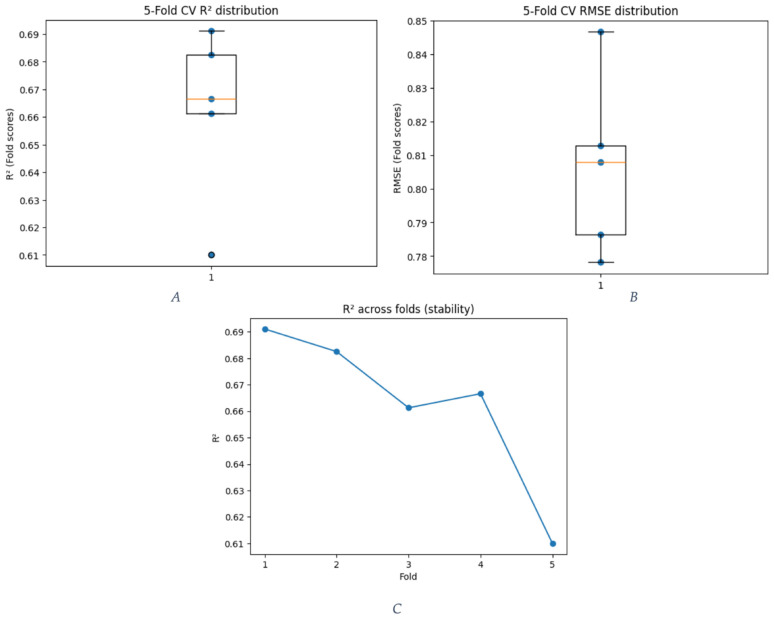
Cross-validation performance analysis of the optimized regression framework. (**A**) Distribution of 5-fold cross-validation R^2^ values, (**B**) distribution of 5-fold RMSE values, and (**C**) fold-dependent R^2^ stability analysis across independent validation subsets. The orange horizontal line in panels (**A**) and (**B**) represents the median value of the corresponding cross-validation metric.

To further evaluate the robustness, reproducibility, and predictive stability of the developed regression framework, a 5-fold cross-validation procedure was applied to the optimized HistGradientBoostingRegressor model [18,24,25]. During cross-validation, the curated FXIa dataset was repeatedly partitioned into independent training and validation subsets, and the predictive performances were averaged across all folds to minimize partition-dependent bias and evaluate the reproducibility of the model under multiple independent validation conditions. The 5-fold cross-validation analysis yielded a mean Q^2^ value of approximately 0.66 ± 0.03, whereas the corresponding RMSEcv value remained approximately 0.81 ± 0.02. The relatively limited variation observed between the validation folds further supports the robustness and reproducibility of the optimized regression framework across independent data partitions [18]. Cross-validation analysis demonstrated a stable predictive behavior across different validation folds. The mean cross-validation R^2^ value remained close to the independent test set performance, whereas the RMSE values exhibited relatively low variation between folds. The narrow distribution observed in both the R^2^ and RMSE boxplot analyses indicated that the developed regression framework maintained a consistent predictive capability across multiple independent data partitions. The fold-dependent R^2^ distribution further demonstrated that the predictive performance remained relatively stable throughout the validation process, despite minor variations associated with dataset partitioning. These findings suggest that the developed QSAR framework was not strongly dependent on a specific train–test split and exhibited acceptable generalization capability across the curated FXIa chemical space [26,27]. To improve algorithmic robustness and ensure fair model comparison, hyperparameter optimization was subsequently performed using the RandomizedSearchCV framework implemented in Scikit-learn. Multiple combinations of learning rate parameters, tree depth configurations, leaf node structures, iteration numbers, and regularization parameters were systematically evaluated using 5-fold cross-validation exclusively on the training dataset. The optimized HistGradientBoostingRegressor model achieved a predictive performance comparable to the baseline ensemble learning configuration, with an independent test-set R^2^ value of approximately 0.71 and an RMSE value near 0.76. The relatively limited performance variation observed after optimization suggests that the selected ensemble-learning architecture was intrinsically well-suited for modeling the curated FXIa descriptor space and that the predictive behavior was not solely dependent on aggressive parameter tuning. As illustrated in Figure 1, the optimized regression framework exhibited stable predictive behavior across independent validation folds with relatively limited variation in both R^2^ and RMSE distributions. The fold-dependent stability analysis confirmed the reproducibility and robustness of the developed QSAR framework across multiple independent validation subsets [18].

Overall, the cross-validation and hyperparameter optimization analyses demonstrated that the developed regression framework exhibited stable predictive behavior, acceptable reproducibility, and robust generalization capability across structurally diverse FXIa inhibitors.

### 2.4. Y-Randomization Analysis

**Figure 2:**
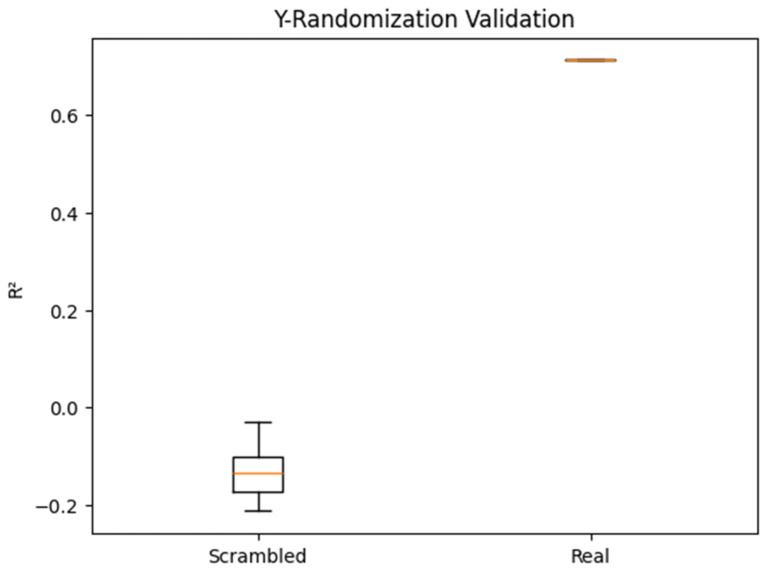
Y-randomization validation analysis of the optimized regression framework. The orange horizontal line in the boxplot represents the median R^2^ value.

To evaluate the possibility of chance correlations and further validate the scientific reliability of the developed QSAR framework, Y-randomization analysis was performed using randomly shuffled pKi values while preserving the original descriptor matrix and the preprocessing workflow [18,28]. The regression models were subsequently retrained using the identical machine learning procedures applied to the randomized datasets. The randomized models exhibited substantially lower predictive performance than the original regression framework. In particular, the scrambled datasets produced strongly reduced and frequently negative R^2^ values, indicating the absence of meaningful predictive relationships after the disruption of the original descriptor–activity associations [29]. The distribution of the randomized R^2^ values was significantly separated from the real model performance, confirming that the predictive capability of the developed QSAR framework did not arise from random statistical correlations. A comparison between the randomized and real-model predictive performances is illustrated in Figure 2.

The randomized models exhibited substantially lower predictive performance than the original model, confirming that the observed QSAR performance did not arise from chance correlations [28,29]. Similarly, the RMSE values obtained from the randomized models were considerably higher than those observed for the original optimized regression framework. A comparison between the real and randomized prediction distributions clearly demonstrated that the experimentally observed predictive performance could not be reproduced after target randomization [29]. The Y-randomization histograms and validation plots further illustrate the strong separation between the real model performance and the scrambled model distributions. While the original model achieved stable predictive performance with a test-set R^2^ value near 0.71, the randomized models clustered around substantially lower predictive values, confirming that the machine learning framework captured meaningful structure–activity relationships within the curated FXIa dataset rather than random mathematical associations [30,31,32]. Overall, the Y-randomization analysis provided strong evidence supporting the robustness, reliability, and scientific validity of the developed regression framework and reduced the likelihood that the observed predictive performance resulted from chance correlations or overfitting [18,33].

### 2.5. Applicability Domain Analysis

**Figure 3:**
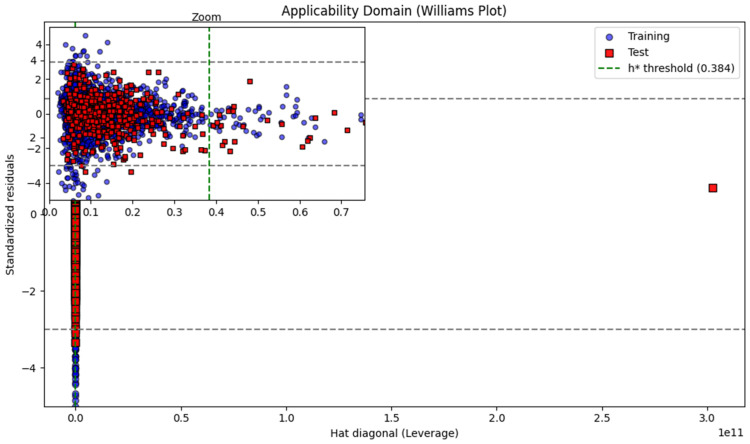
Applicability domain analysis of the optimized regression framework using the leverage-based Williams plot approach.

The applicability domain (AD) of the developed regression framework was evaluated using the leverage-based Williams plot approach to assess prediction reliability and identify structurally influential compounds within the curated FXIa dataset [29,34]. Williams plots enabled simultaneous visualization of leverage values and standardized residual distributions for both the training and independent test compounds. Most compounds were distributed within the accepted applicability domain boundaries, indicating that most predictions were generated within the reliable chemical space represented by the training dataset [18]. Only a limited number of compounds exhibited elevated leverage values or standardized residuals outside the accepted threshold limits, suggesting the presence of structurally influential molecules or prediction outliers in the dataset [35]. The leverage distributions demonstrated that the descriptor space remained relatively stable and that the curated dataset did not contain excessive numbers of highly influential compounds capable of dominating the regression behavior [34]. Furthermore, most standardized residual values remained within the accepted ±3 residual interval, indicating acceptable predictive consistency throughout the training and independent validation datasets. Williams plot analysis additionally suggested that the developed QSAR framework maintained reliable predictive performance across a broad region of the explored FXIa chemical space. Although several compounds displayed higher leverage behavior because of their structural uniqueness or increased molecular complexity, these molecules did not substantially compromise the overall predictive stability of the regression model [30]. The leverage-based applicability domain analysis obtained using the Williams plot approach is shown in Figure 3.

Most compounds remained within the accepted leverage and standardized residual thresholds, indicating reliable predictive behavior across the explored FXIa chemical space. Therefore, the applicability domain analysis confirmed that the developed machine-learning framework generated predictions primarily within a chemically meaningful and statistically reliable descriptor space. These findings further support the robustness and practical applicability of the proposed FXIa QSAR models for structurally diverse inhibitor classes.

### 2.6. Experimental Versus Predicted pKi Analysis

**Figure 4:**
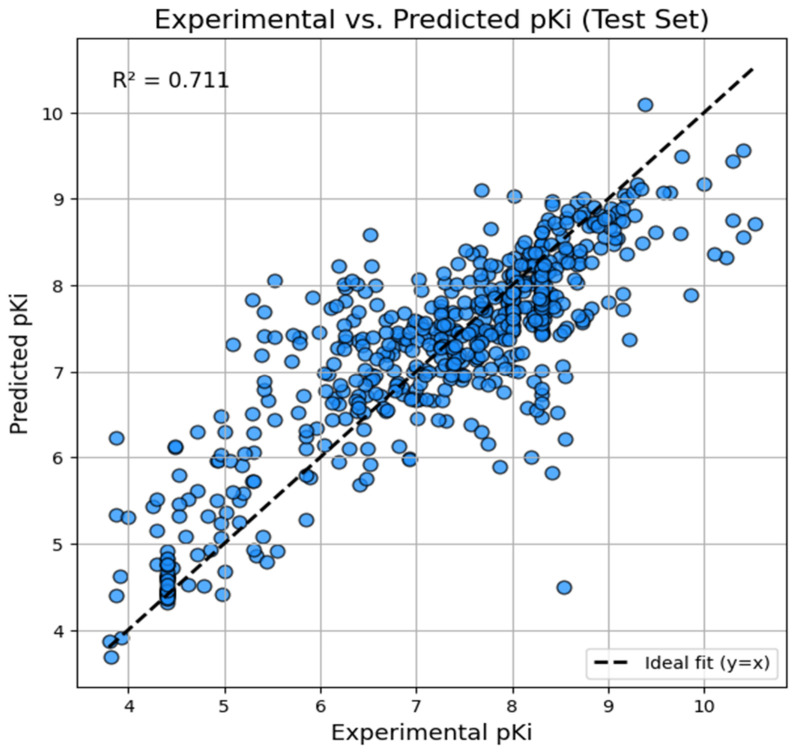
Experimental versus predicted pKi values obtained for the independent test dataset using the optimized HistGradientBoostingRegressor model.

The predictive capability of the optimized regression framework was further evaluated by comparing the experimentally observed pKi values with the model-predicted activities for the independent test dataset [36,37]. Scatter plot analysis demonstrated a generally strong agreement between the predicted and experimental values, with most compounds distributed near the diagonal reference line, corresponding to ideal prediction behavior [38]. The optimized HistGradientBoostingRegressor model achieved an independent test-set R^2^ value of approximately 0.71, with an RMSE value near 0.76, indicating acceptable predictive accuracy across structurally diverse FXIa inhibitors. The relatively high R^2^ value suggests that the developed regression framework successfully captured a substantial proportion of the variance associated with FXIa inhibitory activity in the curated descriptor space [34,37]. Although several compounds exhibited moderate deviations from the ideal prediction line, the overall prediction distribution remained balanced, without evidence of systematic overprediction or underprediction. Larger prediction deviations were observed primarily for compounds located at the extremes of the activity distribution, which are commonly encountered in heterogeneous medicinal chemistry datasets containing structurally diverse inhibitor classes [33]. The observed predictive behavior additionally supports the suitability of ensemble-based machine learning architectures for modeling nonlinear structure–activity relationships associated with FXIa inhibition in silico. The relatively compact distribution of the prediction residuals suggests that the selected descriptor successfully captured the key physicochemical and structural properties relevant to ligand activity prediction [36]. The relationship between the experimentally observed and model-predicted pKi values for the independent test dataset is shown in Figure 4.

Most compounds were distributed near the diagonal reference line, indicating an acceptable predictive agreement between the observed and predicted FXIa inhibitory activities. Overall, the agreement between the experimental and predicted pKi values demonstrated that the developed regression framework exhibited acceptable predictive performance, robust generalization behavior, and reliable applicability across the curated FXIa inhibitor dataset.

### 2.7. SHAP-Based Mechanistic Interpretation of FXIa Inhibition

**Figure 5:**
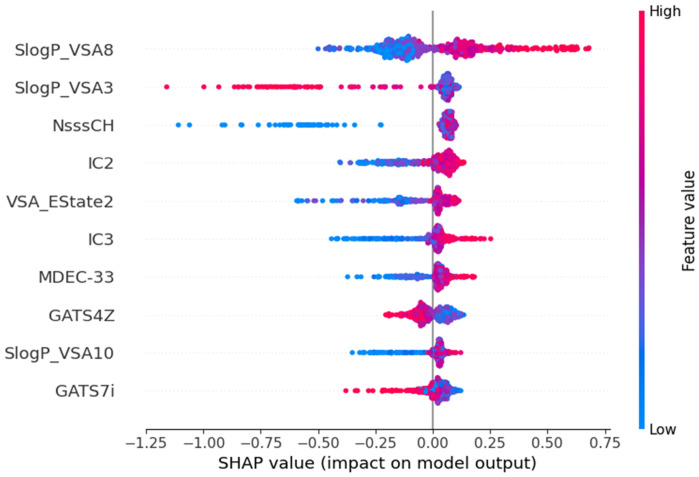
SHAP summary plot illustrating the contributions of the most influential molecular descriptors to FXIa inhibitory activity prediction.

To improve interpretability and identify the molecular features most strongly associated with FXIa inhibitory activity, SHAP-based explainable artificial intelligence analysis was performed for the optimized regression and classification frameworks [37]. The SHAP summary plots revealed that descriptors associated with lipophilicity, aromaticity, electrostatic surface distribution, molecular topology, and drug-likeness exerted a dominant influence on the predictive behavior of the developed models [36]. Among the most influential descriptor groups, SlogP_VSA-related descriptors demonstrated strong contributions to model predictions, indicating that the hydrophobic surface area and lipophilicity played critical roles in determining FXIa inhibitory potency. This observation is chemically reasonable because the FXIa active site contains hydrophobic subpockets capable of stabilizing aromatic and lipophilic ligand frameworks through favorable non-polar interactions [39]. Similarly, the PEOE_VSA descriptors associated with the partial charge distribution and electrostatic surface properties also contributed substantially to the predictive framework. These findings suggest that the electrostatic complementarity between the inhibitors and FXIa binding cavity is an important determinant of ligand recognition and binding stability [40]. The simultaneous importance of hydrophobic and electrostatic descriptors indicates that successful FXIa inhibitors require balanced physicochemical properties rather than simple hydrophobic optimization [41]. The SHAP-based descriptor importance distributions obtained using the optimized regression framework are shown in Figure 5.

Lipophilicity-related, electrostatic, and topological descriptors exhibited dominant contributions to the predictive behavior of the optimized regression framework [36]. Ring-related descriptors and topological complexity indices also emerged as dominant contributors to regression and classification analyses. The importance of these descriptors suggests that the organization of the aromatic scaffold and molecular topology strongly influence ligand accommodation within the FXIa active site [12]. These observations are consistent with the structural characteristics of known FXIa inhibitors, which frequently contain heteroaromatic systems and conformationally constrained scaffold architectures that are optimized for selective target binding. The SHAP-derived descriptor patterns were consistent with those of previously reported FXIa inhibitors, such as milvexian and asundexian, both of which contain hydrophobic heteroaromatic frameworks designed to optimize interactions within the S1 and S2 binding pockets of FXIa [42,43]. The observed importance of lipophilicity- and topology-related descriptors supports the role of aromatic stacking interactions, hydrophobic complementarity, and pocket occupancy in stabilizing FXIa inhibitor binding. Overall, SHAP analysis provided mechanistic insights into the molecular determinants governing FXIa inhibitory activity and demonstrated that the developed machine learning framework captured chemically meaningful structure–activity relationships rather than relying solely on abstract mathematical correlations [37].

### 2.8. Classification Modeling and Predictive Performance

In addition to continuous activity prediction, a binary classification framework was developed to distinguish between active and inactive FXIa inhibitors based on experimentally derived pKi thresholds. The initial classification dataset comprised 3011 compounds. To reduce label ambiguity and improve the reliability of binary classification, intermediate-activity compounds were excluded according to predefined activity thresholds. Following this filtering procedure, the final binary classification dataset used for model development consisted of 2119 compounds. Compounds with pKi values greater than 7.5 were classified as active, whereas those with pKi values < 6.0 were classified as inactive. The benchmarking analysis demonstrated that the ensemble-based classification algorithms consistently outperformed the conventional linear classifiers across all evaluated performance metrics. Tree-based ensemble architectures, including ExtraTreesClassifier, RandomForestClassifier, and gradient-boosting approaches, exhibited particularly strong predictive capabilities for distinguishing highly active FXIa inhibitors from weakly active compounds. Among the evaluated classifiers, the optimized ensemble-based classification framework achieved high predictive accuracy and strong ROC-AUC performance, indicating robust discrimination capability between active and inactive inhibitor classes. A comparative benchmarking summary of the top-performing classification algorithms is provided in Table 2.

**Figure 6:**
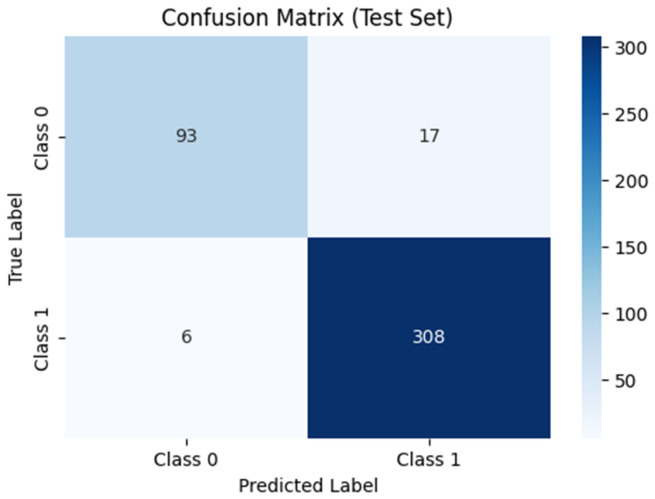
Confusion matrix obtained for the independent test dataset using the optimized ensemble-based classification framework.

The ranking of the classification algorithms presented in Table 2 was primarily based on overall balanced classification performance by collectively considering Accuracy, F1-score, and ROC-AUC metrics rather than ROC-AUC values alone. The strong predictive performance observed even for conventional linear classifiers, such as LogisticRegression and LinearSVC, suggests that the curated descriptor space retained meaningful discriminatory information associated with FXIa inhibitory activity. Nevertheless, ensemble-based classifiers consistently demonstrated superior predictive robustness and ROC-AUC performance, supporting the suitability of nonlinear ensemble learning approaches for FXIa activity classification. The confusion matrix analysis additionally demonstrated a balanced classification behavior with relatively low false-positive and false-negative prediction rates. The classification performance of the optimized ensemble-based framework on the independent test dataset is illustrated in Figure 6.

The model demonstrated balanced classification behavior with relatively low false-positive and false-negative prediction rates for active and inactive FXIa inhibitors [44,45]. Cross-validation analysis further confirmed the stability of our classification framework. The mean cross-validation accuracy remained approximately 0.91, with a relatively low standard deviation across independent folds, indicating that the classification performance was not strongly dependent on a specific training–test partition [38]. These findings suggest that the developed classifier exhibits acceptable generalization capability throughout the curated FXIa chemical space [46]. The classification Y-randomization analysis additionally demonstrated a substantial reduction in predictive discrimination capability after activity label shuffling [28]. Although partial classification accuracy remained because of class imbalance effects, the randomized ROC-AUC values decreased markedly compared with those of the original model, confirming that the classification framework captured meaningful structure–activity relationships rather than random statistical patterns [30,45]. The strong performance of ensemble-based classifiers additionally supports the suitability of nonlinear machine learning approaches for modeling heterogeneous medicinal chemistry datasets containing structurally diverse FXIa inhibitor families. Overall, the developed classification framework demonstrated reliable predictive behavior and strong applicability for distinguishing between active and inactive FXIa inhibitor chemotypes [37].

### 2.9. Overall Evaluation of the Developed QSAR Framework

Collectively, the integration of benchmarking analysis, scaffold-aware validation, applicability domain assessment, 5-fold cross-validation, Y-randomization testing, classification modeling, and SHAP-based explainable artificial intelligence substantially improved the scientific reliability, robustness, and interpretability of the developed FXIa-focused QSAR framework [21,46,47,48]. Among the evaluated machine learning approaches, nonlinear-ensemble learning consistently outperformed conventional linear models, indicating that FXIa inhibitory activity depends on multidimensional and nonlinear relationships among physicochemical, topological, aromatic, electrostatic, and lipophilicity-associated molecular properties [49,50]. The optimized HistGradientBoostingRegressor framework demonstrated the most balanced predictive behavior across independent validation analyses and provided the best compromise between the predictive capability and generalization performance [38]. Scaffold-aware validation analysis further demonstrated the increased difficulty associated with extrapolating chemically distinct FXIa inhibitor chemotypes. Under scaffold-separated validation conditions, the predictive performance decreased to an R^2^ value of approximately 0.19, with an RMSE value near 0.86, reflecting the substantial structural dissimilarity between the scaffold-separated training and test datasets. Nevertheless, considering the chemically heterogeneous nature of the curated FXIa inhibitor dataset, the retained predictive capability still suggests the presence of transferable structure–activity relationship (SAR) patterns beyond simple scaffold memorization. The applicability domain analysis demonstrated that most predictions remained within statistically reliable descriptor space boundaries, whereas Y-randomization testing confirmed that the predictive performance did not arise from random statistical correlations or from mathematical artifacts. In parallel, the SHAP-based mechanistic interpretation revealed that descriptors associated with lipophilicity, aromatic scaffold organization, electrostatic surface distribution, halogen-related properties, drug-likeness filters, and molecular topology collectively exerted dominant influences on the FXIa-inhibitory activity prediction [49,50,51]. The observed descriptor-importance patterns were consistent with the reported structural characteristics of clinically investigated FXIa inhibitors, including milvexian and asundexian, which contain hydrophobic heteroaromatic frameworks optimized for favorable interactions within the S1 and S2 binding pockets of FXIa. These findings further support the ability of the developed machine learning framework to capture chemically meaningful structure–activity relationships relevant to medicinal chemistry optimization. Overall, the combined integration of regression modeling, 5-fold cross-validation, classification analysis, benchmarking-driven model selection, applicability domain assessment, scaffold-aware validation, Y-randomization validation, and explainable artificial intelligence substantially improved the robustness, interpretability, reproducibility, and scientific reliability of the developed quantitative structure–QSAR framework [28,34,35,36,38,45].

### 2.10. Proof-of-Concept Virtual Screening

To further evaluate the practical applicability of the developed machine learning framework for FXIa-focused drug discovery, a proof-of-concept virtual screening analysis was performed using intermediate-activity compounds that were previously excluded from the binary classification framework [46,47,48,49,50,51]. Compounds with experimental pKi values between 6.0 and 7.5 were selected as a chemically relevant screening library to evaluate the prioritization capability of the developed regression and classification models under uncertain activity conditions. The curated screening compounds were processed using the identical descriptor generation, feature selection, and preprocessing pipeline employed during model development [21]. The optimized HistGradientBoostingRegressor model was subsequently used for quantitative pKi prediction, and the optimized classification framework was applied to estimate the probability of FXIa inhibitory activity for each screened compound.

The virtual screening analysis identified multiple compounds exhibiting high predicted pKi values and elevated active-class probabilities. Several top-ranked candidate compounds demonstrated predicted pKi values exceeding 8.0, with active probabilities approaching 1.0, suggesting a favorable predicted FXIa inhibitory potential according to both regression- and classification-based prioritization strategies [45,49]. A summary of the top-ranked candidate compounds identified during the proof-of-concept virtual screening is presented in Table 3.

Importantly, the combined integration of quantitative regression prediction and classification-based activity prioritization enabled the identification of promising FXIa inhibitor candidates from structurally heterogeneous intermediate-activity compounds. These findings further support the practical utility of the developed QSAR framework for future ligand prioritization, virtual screening campaigns, and medicinal chemistry optimization studies targeting FXIa-associated anticoagulant drug discovery [19,47,52,53,54,55,56].

## 3. Materials and Methods

**Figure 7:**
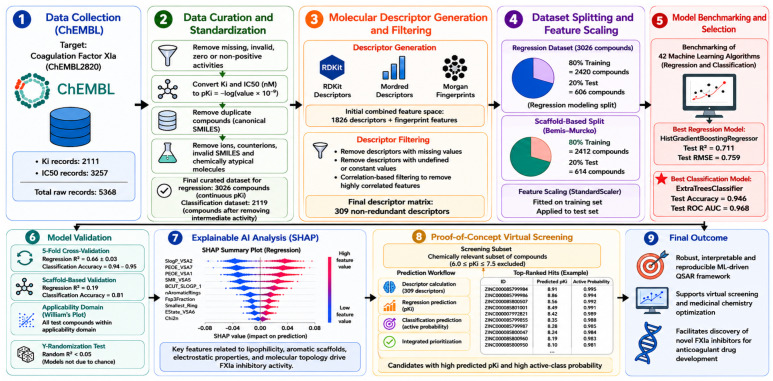
Schematic representation of the integrated machine learning workflow developed for predicting FXIa inhibitory activity.

The overall workflow of the developed machine learning framework is illustrated in Figure 7. The workflow included ChEMBL-based data retrieval from the ChEMBL database (European Bioinformatics Institute, Hinxton, UK), molecular curation and standardization, descriptor generation using RDKit (version 2026.3.2, RDKit Project, available at https://www.rdkit.org) and Mordred (version 1.2.0, available at https://github.com/mordred-descriptor/mordred, accessed on 1 May 2026), descriptor filtering, dataset splitting and feature scaling, benchmarking-driven model selection using machine learning algorithms implemented in Scikit-learn (version 1.6.1, available at https://scikit-learn.org), validation procedures including cross-validation, scaffold-based analysis using the Bemis–Murcko scaffold approach implemented in RDKit, and SHAP-based interpretability assessment. Additional data processing and analysis were performed using Python (version 3.12, Python Software Foundation, Wilmington, DE, USA), Pandas (version 2.2.2, available at https://pandas.pydata.org), and NumPy (version 2.0.2, available at https://numpy.org). Detailed information regarding all software packages, version numbers, and corresponding sources is provided in the Section 3. Additional supporting analyses and methodological details are provided in the Supplementary Materials.

### 3.1. Data Collection and Bioactivity Retrieval

Bioactivity data for small-molecule Coagulation Factor XIa (FXIa) inhibitors were retrieved from the ChEMBL database using the ChEMBL Web Resource Client Python API (version 0.10.9; European Bioinformatics Institute, Hinxton, UK).Target identification was performed using the keyword “Coagulation Factor XIa”, and the corresponding ChEMBL target identifier (CHEMBL2820) was selected for data retrieval. Only compounds with experimentally determined inhibitory activities were retained for subsequent analyses. Specifically, inhibitory constant (Ki) and half-maximal inhibitory concentration (IC_50_) records were collected, as these endpoints provide quantitative bioactivity information suitable for ligand-based QSAR modeling. The initial query retrieved 2111 Ki records and 3257 IC_50_ records. After combining both datasets, an initial raw bioactivity dataset comprising 5368 entries was obtained, including molecular identifiers, canonical SMILES strings, activity values, and assay-related metadata.

### 3.2. Activity Standardization and Molecular Data Curation

The raw bioactivity dataset was subjected to a multistep curation procedure prior to the descriptor calculation and machine learning analysis. Records with missing, invalid, zero, or non-positive activity values were excluded from the study. Activity values reported in nanomolar units were transformed into a negative logarithmic scale according to the following equation:

pKi = −log_10_ (Activity × 10^−9^) (1)

where Activity represents the experimentally reported Ki or IC_50_ value in nanomolar concentration. For consistency throughout the study, the transformed activity endpoint was referred to as pKi. Duplicate compounds were identified using the canonical SMILES representations. After removing duplicate molecular structures, the dataset was reduced from 5368 raw records to 3637 unique compounds. Additional molecular standardization steps were applied, including the removal of invalid SMILES, missing structural information, ionic/counterion-containing structures, and chemically atypical molecules from the dataset. In addition, Lipinski’s rule-of-five filtering was applied to remove compounds exhibiting unfavorable drug-like properties and improve the chemical consistency of the curated dataset [57]. After ion removal, Lipinski filtering, and structural preprocessing, the curated dataset contained 3523 structurally valid compounds. Continuous activity-based filtering was subsequently applied by retaining compounds with valid continuous pKi values within the selected modeling range for machine-learning analysis. The final regression dataset comprised 3026 compounds.

### 3.3. Molecular Descriptor Generation and Feature Filtering

Molecular descriptors were generated using RDKit, fingerprint representations, and the Mordred descriptor calculation package (https://github.com/mordred-descriptor/mordred, accessed on 1 May 2026) [58,59]. Canonical SMILES structures were converted into RDKit molecular objects prior to descriptor computation. Initially, RDKit-based descriptors were calculated to obtain the physicochemical, constitutional, and topological molecular properties. Morgan fingerprints were also explored during the preliminary descriptor analyses. In parallel, Mordred descriptors were computed to generate a comprehensive descriptor library, including constitutional, topological, geometrical, electronic, and physicochemical molecular features. For the Mordred descriptor generation, three-dimensional molecular conformations were constructed using RDKit embedding procedures. Molecules for which conformer generation or descriptor calculation failed because of structural instability were excluded from the subsequent analyses. The initial Mordred descriptor matrix consisted of approximately 1826 molecular descriptors. A multistep descriptor-filtering workflow was subsequently applied to improve the descriptor quality and reduce feature redundancy. Initially, descriptors containing missing values, undefined outputs or constant values were removed. Correlation-based filtering was performed to eliminate highly correlated descriptors and reduce multicollinearity within the descriptor matrix. Following descriptor pre-processing and redundancy reduction, the final regression descriptor matrix consisted of approximately 309 non-redundant Mordred descriptors representing 3026 curated FXIa inhibitors. Although RDKit descriptors and Morgan fingerprints were evaluated during exploratory analyses, the final QSAR framework was primarily constructed using the filtered Mordred descriptor matrix because of its improved interpretability and compatibility with explainable artificial intelligence analyses [59,60,61].

### 3.4. Dataset Splitting and Feature Scaling

Prior to the machine learning analysis, the final regression descriptor matrix was divided into independent training and test subsets using an 80:20 random split. The resulting dataset partitions consisted of approximately 2420 training compounds and 606 independent test compounds in total. The descriptor values were standardized using the StandardScaler method implemented in the scikit-learn framework. To prevent information leakage during model development, the scaling parameters were fitted exclusively using the training dataset and were subsequently applied to the independent test dataset using identical transformation coefficients. In addition to conventional random splitting, scaffold-aware validation was performed using Bemis–Murcko scaffold decomposition to evaluate model generalizability across chemically distinct molecular frameworks. Molecular scaffolds were generated from canonical SMILES structures using RDKit scaffold decomposition procedures. Compounds with identical scaffolds were grouped together during dataset partitioning to prevent structurally similar molecules from appearing simultaneously in both the training and test datasets. The scaffold-aware validation dataset consisted of approximately 2412 training compounds and 614 test compounds, which represented chemically dissimilar scaffold distribution [59,60,61]. A total of 1186 unique molecular scaffolds were identified in the curated FXIa dataset used in this study. This strategy enabled the evaluation of the model’s extrapolation capability across structurally distinct chemotypes.

### 3.5. Model Evaluation Metrics

To evaluate the predictive performance of the developed machine learning models, standard regression and classification metrics were employed. For the regression analyses, the coefficient of determination (R^2^) and root mean square error (RMSE) were calculated using the following equations:

(2) $$R^{2}=1-\frac{\sum (y_{i}-{\hat{y}}_{i}{)}^{2}}{\sum (y_{i}-\bar{y}{)}^{2}}$$

(3) $$RMSE=\sqrt{\frac{\sum (y_{i}-{\hat{y}}_{i}{)}^{2}}{n}}$$

where $y_{i}$ represents the experimentally observed pKi value, ${\hat{y}}_{i}$ denotes the predicted pKi value, $\bar{y}$ corresponds to the mean experimental activity, and n represents the total number of compounds. The R^2^ metric reflects the proportion of variance explained by the model, whereas the RMSE quantifies the average prediction error between the experimental and predicted values [42]. For the classification analyses, the predictive performance was evaluated using Accuracy, Precision, Recall, F1-score, and receiver operating characteristic area under the curve (ROC-AUC) metrics derived from the confusion matrix:

(4) $$Accuracy=\frac{TP+TN}{TP+TN+FP+FN}$$

(5) $$Precision=\frac{TP}{TP+FP}$$

(6) $$Recall=\frac{TP}{TP+FN}$$

(7) $$\text{F1-score}=2\times \frac{Precision\times Recall}{Precision+Recall}$$

where TP, TN, FP, and FN represent true positives, true negatives, false positives, and false negatives, respectively. For regression modeling, the curated dataset consisting of 3026 compounds was randomly divided into training and independent test subsets using an 80:20 split. Similarly, for classification modeling, the filtered dataset containing 2119 compounds was partitioned using the same 80:20 training–test strategy after excluding intermediate-activity compounds. All predictive metrics were calculated using an independent test dataset to objectively evaluate the generalization capability of the developed machine learning frameworks.

### 3.6. Benchmarking and Machine Learning Modeling

To identify the most suitable machine learning algorithm for predicting FXIa inhibitory activity, a comprehensive benchmarking analysis was performed using the LazyPredict framework. Multiple regression algorithms were systematically evaluated under identical preprocessing conditions, descriptor sets, and training–test partitions. The model performance was evaluated using the coefficient of determination (R^2^) and the root mean square error (RMSE). Benchmarking analysis demonstrated that ensemble-based machine learning algorithms consistently outperformed conventional linear models in predicting FXIa inhibitory potency. Among the evaluated algorithms, the HistGradientBoostingRegressor demonstrated the highest predictive performance, with an independent test-set R^2^ value of approximately 0.71 and an RMSE of approximately 0.76. Additional high-performing models included the LGBMRegressor, ExtraTreesRegressor, Support Vector Regression (SVR), and RandomForestRegressor. Conventional linear regression approaches, such as LinearRegression, Ridge, and BayesianRidge, exhibited substantially lower predictive performance compared to nonlinear ensemble-based methods. A comparison between linear and nonlinear algorithms demonstrated that nonlinear machine learning architectures were more effective in capturing the complex structure–activity relationships associated with FXIa inhibition than linear algorithms [57,60,61,62].

### 3.7. Hyperparameter Optimization

To improve the predictive robustness and ensure a fair algorithmic comparison, hyperparameter optimization was performed for the selected regression models using the RandomizedSearchCV framework implemented in scikit-learn. Optimization procedures were conducted using 5-fold cross-validation exclusively on the training dataset to prevent information leakage from the independent test set. Multiple parameter combinations controlling the learning behavior, tree complexity, leaf node structure, and regularization strength were systematically evaluated. For the HistGradientBoostingRegressor model, the optimization workflow included the evaluation of the learning rate, maximum iteration number, maximum tree depth, maximum leaf node count, minimum leaf size, and L2 regularization parameters. The optimized model achieved a predictive performance comparable to the default configuration, indicating that the selected ensemble architecture was intrinsically well-suited for modeling the curated FXIa descriptor space.

### 3.8. Regression Cross-Validation and Y-Randomization Analysis

Model robustness and reproducibility were evaluated using repeated cross-validation and Y-randomization analyses. A 5-fold cross-validation procedure was applied to assess the predictive stability across multiple independent data partitions. During cross-validation, the regression dataset was repeatedly divided into training and validation subsets, and the predictive metrics were averaged across all folds to reduce partition-dependent bias and evaluate the model consistency. To assess the possibility of chance correlations, Y-randomization analysis was performed by randomly shuffling the target pKi values, while preserving the original descriptor matrix. The machine learning models were subsequently retrained using randomized datasets under identical pre-processing and modeling conditions [28]. The substantially lower predictive performance observed after target randomization confirmed that the developed QSAR models captured meaningful structure–activity relationships rather than random statistical associations. This analysis provides additional evidence supporting the reliability and scientific validity of the proposed machine learning framework [28].

### 3.9. SHAP-Based Interpretability Analysis

To improve model interpretability and identify descriptor-level contributions associated with FXIa inhibitory activity, SHapley Additive exPlanations (SHAP) analysis was applied to the final tree-based regression and classification models. SHAP values were calculated using the TreeExplainer framework, which was implemented in the SHAP Python package [62]. Descriptor contributions were quantified based on their influence on the predicted pKi values in the regression modeling and class probability predictions in the classification analyses. Summary SHAP plots were generated to visualize the descriptor importance rankings and directional effects of the descriptor values on the model predictions. SHAP analysis demonstrated that descriptors associated with lipophilicity, aromatic ring distribution, electrostatic surface properties, molecular topology, and drug-likeness played dominant roles in the prediction process [14]. In particular, descriptors such as SlogP_VSA, PEOE_VSA, ring-related descriptors, topological complexity indices, and physicochemical filter descriptors significantly contributed to the model predictions. These findings suggest that hydrophobic interactions, aromatic scaffold organization, electrostatic complementarity, and molecular topology are important determinants of FXIa inhibitory activity. The observed descriptor patterns were consistent with the structural characteristics reported for known FXIa inhibitors, including milvexian, asundexian, and other heteroaromatic inhibitor classes optimized for interactions within the FXIa S1 and S2 binding pockets. The integration of SHAP-based explainable artificial intelligence analysis enabled a mechanistic interpretation of the developed QSAR framework and improved the transparency and interpretability of the machine learning predictions.

### 3.10. Classification Modeling

In addition to regression modeling, a binary classification framework was developed to distinguish between active and inactive FXIa inhibitors. Compounds with pKi values greater than 7.5 were labeled as active, whereas those with pKi values lower than 6.0 were labeled as inactive. Molecules within the intermediate activity range (6.0 ≤ pKi ≤ 7.5) were excluded from the classification workflow to reduce class ambiguity and improve model robustness. The final classification dataset consisted of approximately 1569 active and 550 inactive compounds in total. Descriptor generation, descriptor filtering, scaling procedures, and dataset preprocessing were performed using the same workflow as that used in the regression analyses. The classification descriptor matrix was divided into independent training and test subsets using an 80:20 split ratio while preserving the class distributions across both datasets. To identify the most suitable machine-learning classifier, a comprehensive benchmarking analysis was performed using the LazyClassifier framework. Multiple classification algorithms were systematically evaluated under identical preprocessing conditions and descriptor sets. Model performance was assessed using accuracy, precision, recall, F1-score, and receiver operating characteristic area under the curve (ROC-AUC). The benchmarking analysis demonstrated that ensemble-based classifiers consistently outperformed conventional linear classification approaches in distinguishing active and inactive FXIa inhibitors. Among the evaluated algorithms, tree-based ensemble models demonstrated the strongest predictive performance and classification stability.

### 3.11. Classification Cross-Validation and Y-Randomization

To evaluate the model robustness, predictive stability, and possibility of chance correlations, cross-validation and Y-randomization analyses were performed on the final classification models. A 5-fold cross-validation procedure was applied to the training dataset to assess predictive consistency across multiple independent partitions. Cross-validation analysis yielded a mean classification accuracy of approximately 0.91 with a low standard deviation, indicating a stable predictive performance and reduced partition-dependent variability. To further evaluate model reliability, Y-randomization analysis was conducted by randomly shuffling the activity labels while preserving the original descriptor matrix. The classification models were subsequently retrained using randomized datasets under identical pre-processing and modeling conditions. Although the classification accuracy remained partially influenced by class imbalance after label randomization, the ROC-AUC value decreased compared to that of the original model. The randomized classification model yielded an ROC-AUC value of approximately 0.59, confirming that the developed classification framework captured meaningful structure–activity relationships rather than purely random statistical patterns. These analyses provide additional evidence supporting the robustness, reproducibility, and scientific validity of the developed FXIa classification models.

### 3.12. Proof-of-Concept Virtual Screening Workflow

To evaluate the practical applicability of the developed machine learning framework for FXIa-focused ligand prioritization, a proof-of-concept virtual screening workflow was performed using intermediate-activity compounds that had been previously excluded from the binary classification framework [60]. Compounds with experimental pKi values between 6.0 and 7.5 were selected as a chemically relevant external screening subset to evaluate the prioritization capability of the developed machine learning models under uncertain activity conditions. The selected screening compounds were processed using identical descriptor generation, preprocessing, and feature selection pipelines employed during regression and classification model development. The descriptor matrix was restricted to the final optimized descriptor subset used during model training to ensure methodological consistency throughout the virtual-screening workflow [60]. For quantitative activity estimation, the optimized HistGradientBoostingRegressor framework was applied to predict the continuous pKi values for all the screened compounds. In parallel, the optimized classification framework was used to estimate the active class probabilities for the same molecular structures. The final prioritization strategy integrated both the predicted pKi values and active-class probabilities to identify compounds exhibiting strong predicted FXIa inhibitory potential and favorable classification confidence. The resulting virtual screening workflow enabled the ranking and prioritization of structurally heterogeneous intermediate-activity compounds based on their predicted FXIa-inhibitory profiles. Top-ranked compounds exhibiting elevated predicted pKi values and high active-class probabilities were selected as proof-of-concept candidate molecules for further computational prioritization [63,64].

## 4. Conclusions

In the present study, a comprehensive and explainable machine learning-driven QSAR framework was developed for modeling Coagulation Factor XIa (FXIa) inhibitory activity using a large curated ChEMBL-derived dataset containing structurally diverse inhibitor chemotypes. The proposed workflow systematically integrates extensive molecular curation, descriptor filtering, benchmarking-based model selection, 5-fold cross-validation, scaffold-aware validation, applicability domain analysis, Y-randomization validation, hyperparameter optimization, and SHAP-based explainable artificial intelligence within a unified predictive framework. The developed regression models demonstrated acceptable predictive capability for quantitative pKi estimation, whereas the complementary classification framework successfully distinguished active and inactive FXIa inhibitors with strong predictive stability and robust performance. The nonlinear ensemble-learning architecture consistently outperformed conventional linear approaches, indicating that FXIa inhibitory activity depends on complex multidimensional relationships among physicochemical, aromatic, electrostatic, topological, and lipophilicity-associated molecular properties. Importantly, scaffold-aware validation analysis demonstrated the inherent difficulty associated with extrapolating chemically distinct FXIa inhibitor scaffolds. Although the predictive performance decreased under scaffold-separated validation conditions compared with conventional random splitting strategies, the developed framework retained a partial predictive capability across chemically dissimilar scaffold distributions. These findings highlight both the robustness of the proposed workflow and the intrinsic challenge of generalizing medicinal machine learning models to structurally novel chemotypes. In parallel, Y-randomization testing confirmed that the predictive behavior did not arise from chance correlations, whereas applicability domain analysis demonstrated that most predictions remained within statistically reliable and chemically meaningful descriptor-space boundaries. The 5-fold cross-validation analysis additionally demonstrated stable predictive behavior with limited performance variation across independent validation folds, further supporting the reproducibility and robustness of the optimized machine learning framework. SHAP-based explainable artificial intelligence analysis further revealed that lipophilicity-associated descriptors, aromatic scaffold organization, electrostatic surface properties, halogen-related descriptors, drug-likeness filters, and molecular topology exerted dominant influences on FXIa inhibitory activity prediction. These observations are consistent with the reported structural characteristics of clinically investigated FXIa inhibitors, such as milvexian and asundexian, particularly regarding hydrophobic interactions and aromatic scaffold accommodation within the S1 and S2 binding pockets of FXIa. To further evaluate the practical applicability of the developed framework, a proof-of-concept virtual screening analysis was performed using intermediate-activity compounds that were previously excluded from the binary classification framework. The integrated regression–classification prioritization strategy successfully identified multiple candidate compounds exhibiting simultaneously high predicted pKi values and elevated active-class probabilities, demonstrating the practical utility of the developed workflow for FXIa-focused ligand prioritization and computational screening. Unlike many previous FXIa QSAR studies that primarily focused on conventional predictive modeling workflows, the present study integrated scaffold-aware validation, applicability domain analysis, Y-randomization validation, explainable artificial intelligence, proof-of-concept virtual screening, and combined regression–classification modeling within a unified interpretable framework. Collectively, these integrated validation, interpretation, and prioritization strategies substantially improved the robustness, interpretability, reproducibility, and scientific reliability of the developed QSAR methodology. Although external prospective validation, structure-based simulations, and experimental screening studies are necessary for future translational applications, the present study provides a reproducible and scientifically robust computational framework that may support future virtual screening campaigns, medicinal chemistry optimization strategies, and the prioritization of structurally diverse FXIa inhibitor candidates for anticoagulant drug discovery.

## Figures and Tables

**Table 1:** Benchmarking results for top-performing regression algorithms.

Rank	Model	Adjusted R^2^	R^2^ (Test)	RMSE (Test)
1	HistGradientBoostingRegressor	0.41	0.711	0.759
2	LGBMRegressor	0.398	0.705	0.767
3	ExtraTreesRegressor	0.393	0.703	0.77
4	SVR	0.386	0.699	0.774
5	NuSVR	0.361	0.688	0.789
6	RandomForestRegressor	0.351	0.683	0.796
7	XGBRegressor	0.332	0.673	0.808
8	GradientBoostingRegressor	0.303	0.659	0.825
9	KNeighborsRegressor	0.248	0.632	0.857
10	BaggingRegressor	0.222	0.619	0.872
11	LassoLarsIC	0.163	0.59	0.904
12	MLPRegressor	0.161	0.59	0.905
13	Ridge	0.158	0.588	0.906
14	RidgeCV	0.155	0.587	0.908
15	BayesianRidge	0.133	0.576	0.92
16	LinearRegression	0.129	0.574	0.922
17	PoissonRegressor	0.107	0.563	0.933
18	ElasticNetCV	0.082	0.551	0.947
19	LassoCV	0.08	0.55	0.948
20	HuberRegressor	0.078	0.549	0.949

**Table 2:** Benchmarking results of the top-performing classification algorithms for FXIa activity classification.

Rank	Model	Accuracy	ROC-AUC	F1-Score
1	ExtraTreesClassifier	0.946	0.968	0.945
2	LogisticRegression	0.941	0.974	0.941
3	XGBClassifier	0.941	0.974	0.94
4	LinearSVC	0.936	0.953	0.936
5	RandomForestClassifier	0.934	0.976	0.932
6	SVC	0.939	0.969	0.937
7	LGBMClassifier	0.934	0.973	0.933

**Table 3:** Top-ranked candidate compounds were identified during the proof-of-concept virtual screening analysis based on the predicted pKi values and active-class probabilities.

Rank	Smiles	pKi	Predicted pKi	Active Probability
1	CN1CCN(c2cccc3c2CCN(C(=O)/C=C/c2c(-n4cnnn4)ccc(Cl)c2F)[C@H]3C(=O)Nc2ccc(C(=O)O)cc2)C(=O)C1	6.5229	8.6779	1.0000
2	CC1CCC[C@H](N2CCC(c3c(C(F)(F)F)ccc(Cl)c3F)=CC2=O)c2cc(ccn2)-c2ccc(C(=O)O)cc2NC1=O	6.4660	8.5179	0.9900
3	CNC(=O)c1ccc(NC(=O)C(CCOC)n2cc(OC)c(-c3cc(Cl)ccc3-n3cc(F)cn3)cc2=O)cn1	7.4318	8.4878	0.9800
4	COC(=O)Nc1ccc(-c2cc([C@H](C[C@@H]3CCCN(C(C)=O)C3)NC(=O)/C=C/c3cc(Cl)ccc3-n3cnnn3)nnc2Cl)cc1	7.0506	8.3319	0.8900
5	COC(=O)Nc1ccc2c(c1)NC(=O)CC(C)CC[C@H](NC(=O)/C=C/c1cc(Cl)ccc1-n1cnnn1)c1nc-2c[nH]1	7.4437	8.3126	0.9800
6	COc1cn(C(CC2CCOC2)C(=O)Nc2ccc(C(=O)O)cc2)c(=O)cc1-c1cc(Cl)ccc1C#N	6.3279	8.1810	0.8500
7	CN(C)C(=O)C1CCN(c2cccc3c2CCN(C(=O)c2cn(-c4cccc(Cl)c4F)nn2)[C@H]3C(=O)Nc2ccc(C(=O)O)cc2)CC1	7.4802	8.1395	1.0000
8	COc1cn(C(CC2CCC(O)CC2)C(=O)Nc2ccc(C(=O)O)cc2)c(=O)cc1-c1cc(Cl)ccc1C#N	6.8861	8.1336	0.6800
9	C[C@@H]1CCC[C@H](N2CCC(c3c(F)ccc(Cl)c3F)=CC2=O)c2cc(ccn2)-c2cc(CO)ccc2NC1=O	7.0453	8.1259	0.9900
10	O=C(O)c1ccc(NC(=O)[C@H]2c3cccc(N4CCC(O)CC4)c3CCN2C(=O)c2cn(-c3cccc(Cl)c3F)nn2)cc1	7.3665	8.1259	1.0000
11	COC(=O)Nc1ccc2c(c1)NC(=O)[C@@H](C)CCC[C@H](N1CCC(c3cccc(Cl)c3F)=CC1=O)c1ccnc-2c1	7.3583	8.1119	0.9900
12	O=C(O)c1ccc(NC(=O)C2c3cccc(C(=O)N4CCNCC4)c3CCN2C(=O)/C=C/c2c(-n3cnnn3)ccc(Cl)c2F)cc1	6.2576	8.0610	0.9900
13	CN(C)C1CCN(c2cccc3c2CCN(C(=O)c2cnn(-c4cccc(Cl)c4F)c2)[C@H]3C(=O)Nc2ccc(C(=O)O)cc2)CC1	7.2636	8.0250	0.9700
14	COCCC(C(=O)Nc1ccc2nc(-c3ccc(F)cc3)cn2c1)n1cc(OC)c(-c2cc(Cl)ccc2C#N)cc1=O	7.4815	8.0245	0.9400
15	COCCC(C(=O)Nc1ccc2nn(C)cc2c1)n1cc(OC)c(-c2cc(Cl)ccc2-c2cocn2)cc1=O	7.4089	8.0239	0.9700
16	C[C@@H]1CCC[C@H](N2CCC(c3c(F)ccc(Cl)c3F)=CC2=O)c2cc(ccn2)-c2ccc(NC(=N)N)cc2NC1=O	7.0410	8.0209	0.9600
17	N[C@H](CF)[C@H]1CC[C@H](C(=O)N2CC[C@H](c3ccccc3)[C@H]2C(=O)Nc2ccc3oc(C(=O)NS(=O)(=O)N4CCOCC4)cc3c2)CC1	6.1612	8.0114	0.8400
18	CCC(C(=O)Nc1ccc2nn(C)cc2c1)n1cc(OC)c(-c2cc(Cl)ccc2-c2cnc(C(F)F)o2)cc1=O	7.1192	7.9927	0.9800
19	CC1(C)Cc2c(cccc2N2CCNCC2)[C@H](C(=O)Nc2ccc(C(=O)O)cc2)N1C(=O)/C=C/c1c(-n2cnnn2)ccc(Cl)c1F	6.9706	7.9864	0.9900
20	COC(=O)Nc1ccc2c(c1)NC(=O)[C@H](C)C(=O)CC[C@H](N1CCC(c3c(F)ccc(Cl)c3F)=CC1=O)c1cc-2ccn1	7.3439	7.9682	0.9900

## Data Availability

The original contributions presented in the study are included in the article and Supplementary Material, the datasets generated and analyzed during the current study are available from the corresponding author on reasonable request.

## Supplementary Materials

The following supporting information can be downloaded at: https://www.mdpi.com/article/10.3390/ph19060912/s1.

## Abbreviations

The following abbreviations are used in this manuscript:

FXIa	Coagulation Factor XIa
QSAR	Quantitative Structure–Activity Relationship
ML	Machine Learning
SHAP	SHapley Additive exPlanations

## References

[1] Palareti G. (2014). Direct Oral Anticoagulants and Bleeding Risk (in Comparison to Vitamin K Antagonists and Heparins), and the Treatment of Bleeding. Semin. Hematol..

[2] Levine M., Goldstein J.N. (2014). Bleeding Complications of Targeted Oral Anticoagulants: What Is the Risk?. Hematology.

[3] Gailani D., Gruber A. (2024). Targeting Factor XI and Factor XIa to Prevent Thrombosis. Blood.

[4] Hsu C., Hutt E., Bloomfield D.M., Gailani D., Weitz J.I. (2021). Factor XI Inhibition to Uncouple Thrombosis From Hemostasis: JACC Review Topic of the Week. J. Am. Coll. Cardiol..

[5] Waisman D.M., Bharadwaj A.G. (2025). Fibrinolysis-Past, Present and Future.

[6] Del Toro-Mijares R., Porres-Aguilar M., Bertoletti L., Tafur A.J., Benzidia I., Cueto-Robledo G., Douketis J.D. (2025). Venous Thromboembolism Prevention and Treatment with Factor XI/XIa Inhibitors: Current Status and Future Perspectives. J. Thromb. Thrombolysis.

[7] Xie Z., Meng Z., Yang X., Duan Y., Wang Q., Liao C. (2023). Factor XIa Inhibitors in Anticoagulation Therapy: Recent Advances and Perspectives. J. Med. Chem..

[8] Xia Y., Hu Y., Tang L. (2023). Factor XIa Inhibitors as a Novel Anticoagulation Target: Recent Clinical Research Advances. Pharmaceuticals.

[9] Cherkasov A., Muratov E.N., Fourches D., Varnek A., Baskin I.I., Cronin M., Dearden J., Gramatica P., Martin Y.C., Todeschini R. (2014). QSAR Modeling: Where Have You Been? Where Are You Going To?. J. Med. Chem..

[10] Todeschini R., Consonni V. (2000). Handbook of Molecular Descriptors.

[11] Gaulton A., Bellis L.J., Bento A.P., Chambers J., Davies M., Hersey A., Light Y., McGlinchey S., Michalovich D., Al-Lazikani B. (2012). ChEMBL: A Large-Scale Bioactivity Database for Drug Discovery. Nucleic Acids Res..

[12] Dragos H., Gilles M., Alexandre V. (2009). Predicting the Predictability: A Unified Approach to the Applicability Domain Problem of QSAR Models. J. Chem. Inf. Model..

[13] Udeabor S.E., Ishfaq M., Shah S.J., Khalid I., Baig F., Hamid M.M.M., Elfadeel A.S.A., Onwuka C.I., Ali S.A.A., Mustafa M.M. (2025). Discovery of Novel FGFR1 Inhibitors for Oral Squamous Cell Carcinoma Using a Multi-Class QSAR Model, Virtual Screening, and Molecular Dynamics Simulations. BMC Cancer.

[14] Schulte L., Ledel B., Herbold S. (2024). Studying the Explanations for the Automated Prediction of Bug and Non-Bug Issues Using LIME and SHAP. Empir. Softw. Eng..

[15] Liang Y., Qiao Z., Meng F. (2022). Identifying TMPRSS2 Inhibitors by Drug Repurposing Screenings of Known FXIa Inhibitors: A Computational Study. Lett. Drug Des. Discov..

[16] Wu J., Yue H., Wang X., Yao Y., Du N., Gong P. (2024). Structure-Based Design and Synthesis of Novel FXIa Inhibitors Targeting the S2’ Subsite for Enhanced Antithrombotic Efficacy. Mol. Divers..

[17] Li Q., Zhang H., Guan S., Du J., Zhang Y., Wang S. (2023). Molecular Dynamics Simulation of the Inhibition Mechanism of Factor XIa by Milvexian-like Macrocyclic Inhibitors. Comput. Theor. Chem..

[18] Tropsha A. (2010). Best Practices for QSAR Model Development, Validation, and Exploitation. Mol. Inform..

[19] Chen H., Engkvist O., Wang Y., Olivecrona M., Blaschke T. (2018). The Rise of Deep Learning in Drug Discovery. Drug Discov. Today.

[20] Sheridan R.P. (2013). Time-Split Cross-Validation as a Method for Estimating the Goodness of Prospective Prediction. J. Chem. Inf. Model..

[21] Wu Z., Ramsundar B., Feinberg E.N., Gomes J., Geniesse C., Pappu A.S., Leswing K., Pande V. (2018). MoleculeNet: A Benchmark for Molecular Machine Learning. Chem. Sci..

[22] Barredo Arrieta A., Díaz-Rodríguez N., Del Ser J., Bennetot A., Tabik S., Barbado A., Garcia S., Gil-Lopez S., Molina D., Benjamins R. (2020). Explainable Artificial Intelligence (XAI): Concepts, Taxonomies, Opportunities and Challenges toward Responsible AI. Inf. Fusion.

[23] Ekins S., Puhl A.C., Zorn K.M., Lane T.R., Russo D.P., Klein J.J., Hickey A.J., Clark A.M. (2019). Exploiting Machine Learning for End-to-End Drug Discovery and Development. Nat. Mater..

[24] Haider I., Li M., Kamran Jamil M. (2026). Graph-Based Stacking Ensemble Approach for Physicochemical Properties Prediction of Oncology-Relevant Compounds. J. Supercomput..

[25] Korlagunta S.R., Selvan I.M., Dhanasekaran S. (2026). Machine Learning-Driven QSAR and Docking Pipeline for Identification of Amyloid Beta-A4 Inhibitors in Alzheimer’s Disease. J. Pharmacol. Pharmacother..

[26] Golbraikh A., Tropsha A. (2002). Beware of Q2!. J. Mol. Graph. Model..

[27] Selassie C.D., Mekapati S.B., Verma R.P. (2002). QSAR: Then and Now. Curr. Top. Med. Chem..

[28] Rücker C., Rücker G., Meringer M. (2007). Y-Randomization and Its Variants in QSPR/QSAR. J. Chem. Inf. Model..

[29] Eriksson L., Jaworska J., Worth A.P., Cronin M.T.D., McDowell R.M., Gramatica P. (2003). Methods for Reliability and Uncertainty Assessment and for Applicability Evaluations of Classification- and Regression-Based QSARs. Environ. Health Perspect..

[30] Tropsha A., Gramatica P., Gombar V.K. (2003). The Importance of Being Earnest: Validation Is the Absolute Essential for Successful Application and Interpretation of QSPR Models. QSAR Comb. Sci..

[31] Eriksson L., Johansson E., Lindgren F., Wold S. (2000). GIFI-PLS: Modeling of Non-Linearities and Discontinuities in QSAR. Quant. Struct.-Act. Relatsh..

[32] Todeschini R., Consonni V. (2009). Molecular Descriptors for Chemoinformatics.

[33] Hawkins D.M. (2003). The Problem of Overfitting. J. Chem. Inf. Comput. Sci..

[34] Gramatica P. (2007). Principles of QSAR Models Validation: Internal and External. QSAR Comb. Sci..

[35] Jaworska J., Nikolova-Jeliazkova N., Aldenberg T. (2005). QSAR Applicability Domain Estimation by Projection of the Training Set in Descriptor Space: A Review. ATLA Altern. Lab. Anim..

[36] Polishchuk P. (2017). Interpretation of Quantitative Structure–Activity Relationship Models: Past, Present, and Future. J. Chem. Inf. Model..

[37] Muratov E.N., Bajorath J., Sheridan R.P., Tetko I.V., Filimonov D., Poroikov V., Oprea T.I., Baskin I.I., Varnek A., Roitberg A. (2020). QSAR without Borders. Chem. Soc. Rev..

[38] Sheridan R.P. (2019). Interpretation of QSAR Models by Coloring Atoms According to Changes in Predicted Activity: How Robust Is It?. J. Chem. Inf. Model..

[39] Al-Horani R.A., Desai U.R. (2014). Recent Advances on Plasmin Inhibitors for the Treatment of Fibrinolysis-Related Disorders. Med. Res. Rev..

[40] Murray C.W., Rees D.C. (2009). The Rise of Fragment-Based Drug Discovery. Nat. Chem..

[41] Lipinski C.A., Lombardo F., Dominy B.W., Feeney P.J. (1997). Experimental and Computational Approaches to Estimate Solubility and Permeability in Drug Discovery and Development Settings. Adv. Drug Deliv. Rev..

[42] Capodanno D., Alexander J.H., Bahit M.C., Eikelboom J.W., Gibson C.M., Goodman S.G., Kunadian V., Lip G.Y.H., Lopes R.D., Mehran R. (2025). Factor XI Inhibitors for the Prevention and Treatment of Venous and Arterial Thromboembolism. Nat. Rev. Cardiol..

[43] Presume J., Ferreira J., Ribeiras R. (2024). Factor XI Inhibitors: A New Horizon in Anticoagulation Therapy. Cardiol. Ther..

[44] Powers D.M.W. (2020). Evaluation: From Precision, Recall and F-Measure to ROC, Informedness, Markedness and Correlation. J. Mach. Learn. Technol..

[45] Gedeck P., Rohde B., Bartels C. (2006). QSAR—How Good Is It in Practice? Comparison of Descriptor Sets on an Unbiased Cross Section of Corporate Data Sets. J. Chem. Inf. Model..

[46] Sinha K., Ghosh N., Sil P.C. (2023). A Review on the Recent Applications of Deep Learning in Predictive Drug Toxicological Studies. Chem. Res. Toxicol..

[47] Jiménez-Luna J., Grisoni F., Schneider G. (2020). Drug Discovery with Explainable Artificial Intelligence. Nat. Mach. Intell..

[48] Jiménez-Luna J., Skalic M., Weskamp N., Schneider G. (2021). Coloring Molecules with Explainable Artificial Intelligence for Preclinical Relevance Assessment. J. Chem. Inf. Model..

[49] Mater A.C., Coote M.L. (2019). Deep Learning in Chemistry. J. Chem. Inf. Model..

[50] Walters W.P., Barzilay R. (2020). Applications of Deep Learning in Molecule Generation and Molecular Property Prediction. Acc. Chem. Res..

[51] Rodríguez-Pérez R., Bajorath J. (2020). Interpretation of Machine Learning Models Using Shapley Values: Application to Compound Potency and Multi-Target Activity Predictions. J. Comput. Aided Mol. Des..

[52] Walters W.P., Murcko M. (2020). Assessing the Impact of Generative AI on Medicinal Chemistry. Nat. Biotechnol..

[53] Schneider G. (2017). Automating Drug Discovery. Nat. Rev. Drug Discov..

[54] Lo Y.C., Rensi S.E., Torng W., Altman R.B. (2018). Machine Learning in Chemoinformatics and Drug Discovery. Drug Discov. Today.

[55] Gawehn E., Hiss J.A., Schneider G. (2016). Deep Learning in Drug Discovery. Mol. Inform..

[56] Baskin I.I., Winkler D., Tetko I.V. (2016). A Renaissance of Neural Networks in Drug Discovery. Expert Opin. Drug Discov..

[57] Kaya A.O. (2026). Interpretable Machine Learning-Driven QSAR Modeling for Coagulation Factor X Inhibitors: From Molecular Descriptors to Predictive Potency. J. Comput. Aided Mol. Des..

[58] Moriwaki H., Tian Y.S., Kawashita N., Takagi T. (2018). Mordred: A Molecular Descriptor Calculator. J. Cheminformatics.

[59] Tosco P., Stiefl N., Landrum G. (2014). Bringing the MMFF Force Field to the RDKit: Implementation and Validation. J. Cheminformatics.

[60] Niazi S.K., Mariam Z. (2023). Recent Advances in Machine-Learning-Based Chemoinformatics: A Comprehensive Review. Int. J. Mol. Sci..

[61] Lin H.H., Han L.Y., Yap C.W., Xue Y., Liu X.H., Zhu F., Chen Y.Z. (2007). Prediction of Factor Xa Inhibitors by Machine Learning Methods. J. Mol. Graph. Model..

[62] Koirala M., Yan L., Mohamed Z., DiPaola M. (2025). AI-Integrated QSAR Modeling for Enhanced Drug Discovery: From Classical Approaches to Deep Learning and Structural Insight. Int. J. Mol. Sci..

[63] Lavecchia A. (2015). Machine-Learning Approaches in Drug Discovery: Methods and Applications. Drug Discov. Today.

[64] Vamathevan J., Clark D., Czodrowski P., Dunham I., Ferran E., Lee G., Li B., Madabhushi A., Shah P., Spitzer M. (2019). Applications of Machine Learning in Drug Discovery and Development. Nat. Rev. Drug Discov..

